# The sGC-stimulator Riociguat for the treatment of Raynaud's phenomenon: A single-dose, double-blind, randomized, placebo-controlled cross-over study (DIGIT)

**DOI:** 10.1186/2050-6511-16-S1-A58

**Published:** 2015-09-02

**Authors:** Michael Huntgeburth, Johannes Kießling, Gerrit Weimann, Verena Kiepsel, Soundos Saleh, Nicolas Hunzelmann, Stephan Rosenkranz

**Affiliations:** 1 Department of Cardiology, Heart Center, University Hospital of Cologne, Cologne, Germany; 2 Bayer Pharma AG, Wuppertal, Germany; 3 Department of Dermatology, University of Cologne, Cologne, Germany

## Background

Raynaud's phenomenon (RP) is a cold- or stress-triggered digital ischemia caused by vasoconstriction in the digital blood vessels, severely affecting patient's life. Medical therapeutic options for RP are limited and commonly unsatisfactory, with a large number of patients not responding to currently available treatment. In the present study we investigated the safety, efficacy and pharmacokinetics of a single dose of the soluble guanylate cyclase (sGC)-stimulator riociguat in patients with RP in a double-blind, randomized, placebo-controlled cross-over fashion.

## Methods and study population

Twenty patients with primary or secondary RP associated with systemic sclerosis (SSc), and symptom duration of ≥1 year, were randomized to receive a single oral dose of 2 mg riociguat or placebo in a cross-over fashion (first visit, 1 week washout-phase, second visit). Patients and investigators were blinded for the study medication at both visits (cross-over). Digital blood flow (DBF) in the right index finger was determined by Laser Speckle Contrast Analysis at room temperature (RT) and 5 minutes after cold water exposure (CWE), both at baseline and 2 hours after study drug administration.

## Results

In the overall study population, riociguat led to an increase in DBF versus baseline of 40.6% at RT and 15.1% after CWE. Based on predefined criteria, patients were considered responders if the placebo-corrected DBF after CWE increased by ≥10% versus baseline at 2 hours after drug intake. This was the case in 12/20 (60%) patients. The highest response rates were seen in patients with primary RP and limited cutaneous SSc, whereas patients with RP associated with diffuse cutaneous SSc responded less well. In responders, riociguat led to an increase in DBF versus baseline of 135.7% at RT and 38.9% after CWE. Riociguat Cmax (±SD) 2 hours after drug intake was 76 ±1.5 μg/ml, which is in the range previously seen in healthy volunteers and patients with pulmonary hypertension. Comparable Cmax values were observed in responders (81 ±1.5 μg/ml) and non-responders (71 ±1.6 μg/ml). AEs were reported in 5 patients receiving riociguat (headache, n=4; dyspepsia, n=1) and 1 patient after placebo intake (malaise). No serious AEs were reported.

## Conclusion

The sGC-stimulator riociguat substantially improved DBF in 60% of patients after cold exposure compared to placebo. It was well tolerated in patients with primary and secondary RP. The results of this study indicate that a longer-term trial investigating the therapeutic effect of riociguat in primary and secondary RP is warranted.

